# Gene Expression Patterns in Peripheral Blood Correlate with the Extent of Coronary Artery Disease

**DOI:** 10.1371/journal.pone.0007037

**Published:** 2009-09-14

**Authors:** Peter R. Sinnaeve, Mark P. Donahue, Peter Grass, David Seo, Jacky Vonderscher, Salah-Dine Chibout, William E. Kraus, Michael Sketch, Charlotte Nelson, Geoffrey S. Ginsburg, Pascal J. Goldschmidt-Clermont, Christopher B. Granger

**Affiliations:** 1 Duke University Medical Center and Duke Clinical Research Institute, Duke University, Durham, North Carolina, United States of America; 2 Novartis Institute for Biomedical Research, Cambridge, Boston, United States of America; 3 UZ Leuven Gasthuisberg, University of Leuven, Leuven, Belgium; Leiden University Medical Center, Netherlands

## Abstract

Systemic and local inflammation plays a prominent role in the pathogenesis of atherosclerotic coronary artery disease, but the relationship of whole blood gene expression changes with coronary disease remains unclear. We have investigated whether gene expression patterns in peripheral blood correlate with the severity of coronary disease and whether these patterns correlate with the extent of atherosclerosis in the vascular wall.

Patients were selected according to their coronary artery disease index (CADi), a validated angiographical measure of the extent of coronary atherosclerosis that correlates with outcome. RNA was extracted from blood of 120 patients with at least a stenosis greater than 50% (CADi≥23) and from 121 controls without evidence of coronary stenosis (CADi = 0).

160 individual genes were found to correlate with CADi (rho>0.2, *P*<0.003). Prominent differential expression was observed especially in genes involved in cell growth, apoptosis and inflammation. Using these 160 genes, a partial least squares multivariate regression model resulted in a highly predictive model (r^2^ = 0.776, *P*<0.0001). The expression pattern of these 160 genes in aortic tissue also predicted the severity of atherosclerosis in human aortas, showing that peripheral blood gene expression associated with coronary atherosclerosis mirrors gene expression changes in atherosclerotic arteries.

In conclusion, the simultaneous expression pattern of 160 genes in whole blood correlates with the severity of coronary artery disease and mirrors expression changes in the atherosclerotic vascular wall.

## Introduction

Coronary artery disease, a multifactorial chronic disease, is the leading cause of death in Western countries. Despite considerable advances in the prevention and treatment of coronary artery disease and its complications, morbidity and mortality remains high. In half of patients with coronary artery disease, the first manifestation is death [1]. Consequently, substantial efforts are being put into the development of new strategies for accurate noninvasive diagnosis of coronary artery disease and the identification of novel treatment targets [2].

Systemic and local inflammation has been shown to play a prominent pathologic role in atherosclerotic coronary artery disease [3]. Adhesion of leukocytes to activated endothelial cells and their migration into the arterial wall are thought to initiate, propagate, and destabilize coronary plaques. All types of blood constituents appear to play a role in plaque formation, although the majority of inflammatory lesions in atherosclerotic vascular tissue consist of foam cell macrophages and activated T-cells [4]. Several studies have found distinct gene expression patterns in atherosclerotic arteries [5]–[8]. While other pathways are likely also important, a consistent feature has been differential expression of inflammatory genes and genes involved in cell cycle control [9]–[12].

Microarray analysis of peripheral blood cells is a practical approach to study gene expression changes that may reflect not only genetic predisposition but also presence and activity of disease, environmental modifier effects, and treatment responses [13]. Total peripheral leukocyte count correlates with the severity of coronary atherosclerosis and is a strong predictor of cardiovascular outcome [14], but little is known about the role of phenotypic changes in circulating blood cells of patients with coronary atherosclerosis. In a recent micro-array analysis, 526 genes were found to be differentially expressed in isolated mononuclear cells from 41 patients [15]. Gene expression patterns of 50 of these genes together with 56 genes selected from the literature were subsequently shown to be associated with the presence of coronary artery disease in two independent cohorts. The aim of the present study was 1) to identify distinct genomic markers in peripheral whole blood that correlate with the severity of coronary artery disease using micro-array analysis and 2) to investigate to what extent gene expression patterns in peripheral blood mirror those in atherosclerotic arteries.

## Methods

### Patient Selection and Characteristics

Patients and control subjects were recruited from individuals that had undergone catheterization in the Duke University Hospital Cardiac Catheterization Laboratory and participated in a proteomics study to discover candidate proteins that are differentially displayed in populations with and those without angiographic coronary artery disease [16]. After being approached and providing informed written consent, subjects had clinical and laboratory data collected. The investigation conforms to the principles outlined in the Declaration of Helsinki, and was approved by the Duke Institutional Review Board.

Patient selection, design and results from the main proteomics study have been reported previously [16]. Populations were initially defined in order to minimize differences in plasma proteins unrelated to the presence or absence of coronary artery disease. As a practical strategy, three different cohorts of subjects (cases and controls) were enrolled: 1) **matched men** (n = 106), who were matched for age and ethnic group, 2) **unmatched men** (n = 82), who did not fulfill the matching criteria and 3) **unmatched women** (n = 53). The severity of coronary artery disease was scored using the Duke Coronary Artery Disease Index (CAD-Index) [17], [18]. The CAD-index is a prognostic assessment of the extent of coronary artery disease, accounting for the number and severity of lesions and diseased vessels and involvement of left anterior descending and left main disease.

Inclusion criteria for the coronary artery disease patient population (cases) were: age between 40 and 65 and coronary artery stenosis of >50% in at least one major coronary artery. Inclusion criteria for the control population (controls) were: age between 40 and 65 for matched men cohort only, no angiographically detectable coronary artery stenosis on cardiac catheterization within the last two years, normal left ventricular ejection fraction and normal regional wall motion. Exclusion criteria for controls were typical signs of angina, or any history or evidence of myocardial ischemia on stress testing, myocardial infarction or unstable angina, any history of peripheral arterial or cerebrovascular disease, or significant vascular stenosis on noninvasive imaging or angiography. Exclusion criteria also included myocardial infarction within one month (for cases), diabetes, uncontrolled hypertension (systolic blood pressure >180 mmHg or diastolic blood pressure >100 mmHg) or with end-organ damage, renal insufficiency (creatinine >2.0 mg/dL or BUN>40 mg/dL), active malignancy, significant valvular heart disease, NYHA Class III or IV heart failure, cigarette smoking >2 packs per day, total cholesterol >300 mg/dL or triglyceride >400 mg/dL, anemia (hemoglobin <12.5 g/dL for females or <13.5 g/dL for males), and hypotension (systolic blood pressure <90 mmHg and diastolic blood pressure <50 mmHg).

### Blood Sampling and Gene Expression Analysis

The blood samples (2.5 mL) were collected in PAXgene™ Blood RNA tubes and total RNA was isolated using the standardized RNA Kit (PreAnalytiX, Qiagen) [19]. RNA isolation started with a centrifugation step to pellet nucleic acids in the PAXgene Blood RNA Tube. The pellet was then washed, and Proteinase K added to digest proteins. Alcohol was added to adjust binding conditions, and the sample was applied to a PAXgene RNA spin column. During a brief centrifugation, RNA selectively bound to the PAXgene silica-gel membrane and eluted using an optimized buffer.

RNA was then quantified by absorbance at A260 nm and the purity was estimated by the ratio A260 nm/A280 nm. RNA integrity was confirmed by non-denaturing agarose gel electrophoresis. RNA was stored at −80°C until further analysis. The quality of 19 RNA samples was insufficient for microarray analysis due to degradation. The genomic studies were conducted in the Novartis Genomics Factory, Basel, Switzerland.

Genome-wide transcript profiling was assessed using human HGU133A oligonucleotide expression probe arrays (Affymetrix, Santa Clara, CA, U.S.A.), comprising 22,483 probe sets. The experiments were done according to the recommendations of the manufacturer [20]. Data was normalized using MAS5 (Affymetrix); the data is publicly available at the Gene Expression Omnibus (GEO) repository (accession number GSE12288, http:/www.ncbi.nlm.nih.gov/geo/query/acc.cgi?acc=GSE12288). As quality control, RT-PCR was performed on 8 selected genes in 2×20 subjects from the ‘matched men’ cohort.

### Independent Evaluation of Predictive Gene Model in Human Aorta Tissue

To test whether the expression pattern in peripheral whole blood is representative for atherosclerosis in general, we have examined the capability of expression of genes derived from the peripheral blood cell study to predict the severity of atherosclerosis in human aortas. Gene expression data was generated using RNA extracted from a unique collection of freshly harvested human aortas with varying degrees of atherosclerosis (n = 67 donors). Donor identification, RNA extraction and micro-array methods (Affymetrix U95Av2) as well as gene expression signatures that differentiate between atherosclerotic disease states in human aortas have been reported previously [8]. As indicated in the original report, disease extent (normal, intermediate, severe) was scored by combining Sudan IV staining and raised lesion data. The “normal” or minimally diseased group showed no Sudan IV staining and contained no raised lesions, while the “intermediate” group showed more than 20% Sudan IV staining but contained no raised lesions. The “severe” group contained raised lesions covering more than 10% of the surface. We identified 20 normal, 25 intermediate and 22 severely diseased sections for this analysis.

### Statistical Methods

Spearman rank correlation between CAD-index and gene expression was calculated (Partek Genomics Suite Version 6.3). An absolute correlation coefficient (rho) >0.2 was considered clinically relevant, corresponding to a p-value of 0.003 (n = 222). Among the 22,483 probe sets of the Affymetrix HGU133A chip, about 60 probe sets can be expected to have an absolute rho>0.2 by chance (false positives). Student's t test, parametric correlation and rank correlation according to Spearman were performed with the statistical software package S-Plus Version 6.

Projections to Latent Structures (PLS) analysis including Orthogonal Signal Correction (OSC) (SIMCA-P Version 10.0) was used to identify gene sets that discriminate between increasing CAD-indices or the three classes (normal, intermediate and severe) of atherosclerosis in the aorta samples. To reduce gene selection bias, models were subsequently repeatedly built based on data from two cohorts to predict CAD in the third cohort. In addition, extensive cross-validation by leave-one-out technique and validation by response permutation was applied to 7 groups of approximately 32 subjects to reduce bias in creating a predictive gene set.

## Results

### Patient Demographics

Demographic data, medical history and medication of the study population are summarized in table 1. A history of hypertension was significantly more common in the cases. Aspirin, statins, and blood pressure lowering agents were more frequently taken by the cases. All controls had no angiographically significant coronary artery disease (CAD-Index = 0). Within the cases, however, there was a wide distribution, with 81% of cases having a CAD-Index between 25 and 63. Although most cases (93%) had at least two-vessel disease or severe single-vessel disease, the distribution of cases is skewed towards the lower end of CAD-Index.

**Table 1:** Demographics and baseline characteristics.

	Matched Men	Unmatched Men	Unmatched Women
	Controls	Cases	P	Controls	Cases	P	Controls	Cases	P
	n = 53	n = 53		n = 38	n = 44		n = 29	n = 24	
Age at time of study (mean±SD)	52±7	53±6	0.77	51±8	58±7	<.001	52±7	54±8	0.27
Age at time of study (median, 25^th^–75^th^)	52 (49–57)	52 (48–57)		50 (46–58)	55 (54–63)		56 (47–56)	54 (50–60)	
Age at last catheterization (mean±SD)	51±6	51±7	0.51	49±8	56±7	<.001	50±7	53±7	0.23
Age at last catheterization (median, 25^th^–75^th^)	50 (47–55)	51 (47–56)		48 (44–57)	54 (52–62)		50 (45–54)	53 (49–57)	
Ethnicity (Caucasian/African-American/Asian/Hispanic/Native Am)	50/3/0/0/0	50/3/0/0/0		23/13/1/1/0	39/4/0/0/1		21/5/0/1/2	18/4/1/0/1	
Smoking, n (%)	26 (49)	29 (55)	0.41	20 (53)	34 (77)	<.001	9 (31)	13 (54)	0.02
Diabetes, n (%)	0 (0)	0 (0)		0 (0)	0 (0)		0 (0)	0 (0)	
Hypertension, n (%)	16 (30)	27 (51)	0.001	15 (39)	27 (61)	<.001	13 (45)	12 (50)	0.30
Myocardial infarction, n (%)	0 (0)	29 (55)		2 (5)	21 (48)	<.001	1 (3)	10 (14)	<.001
PCI, n (%)	0 (0)	15 (28)		0 (0)	13 (30)		0 (0)	4 (20)	
CABG, n (%)	0 (0)	15 (28)		0 (0)	15 (34)		0 (0)	7 (29)	
Peripheral vascular disease, n (%)	0 (0)	2 (4)		0 (0)	5 (11)		0 (0)	3 (13)	
Cerebrovascular disease, n (%)	0 (0)	1 (2)		0 (0)	4 (9)		0 (0)	3 (13)	
Congestive heart failure, n (%)	0 (0)	6 (11)		0 (0)	4 (9)		0 (0)	2 (8)	
Body Weight (kg) (mean±SD)	97±19	94±16	0.40	101±19	94±30	0.27	89±27	84±24	0.43
Systolic blood pressure (mmHg) (mean±SD)	139±19	134±21	0.19	145±17	142±26	0.59	145±23	138±23	0.32
Diastolic blood pressure (mmHg) (mean±SD)	80±11	76±17	0.12	82±10	82±15	0.97	77±12	70±14	0.06
LV ejection fraction (%) (mean±SD)	64±7	56±11	<.001	64±8	57±12	0.004	66±6	57±12	0.001
*Medication*									
Aspirin, n (%)	18 (34)	47 (89)	<.001	10 (26)	42 (95)	<.001	6 (21)	21 (88)	<.001
ACE inhibitor, n (%)	5 (9)	40 (75)	<.001	7 (18)	26 (59)	<.001	4 (14)	13 (54)	<.001
ARB, n (%)	3 (6)	0 (0)	0.07	2 (5)	5 (11)	0.03	0 (0)	5 (21)	
Beta blocker, n (%)	13 (25)	43 (81)	<.001	6 (16)	37 (84)	<.001	6 (21)	20 (83)	<.001
Calcium blocker, n (%)	4 (8)	10 (19)	0.002	5 (13)	9 (20)	0.07	3 (10)	4 (17)	0.19
Statin, n (%)	10 (19)	38 (72)	<.001	4 (11)	29 (66)	<.001	5 (17)	15 (63)	<.001
Fibrate, n (%)	2 (4)	4 (8)	0.15	0 (0)	8 (18)		0 (0)	1 (4)	
*Clinical Laboratory parameters*									
Total Cholesterol (mg/dL) (mean±SD)	196±29	167±32	<0.01	195±39	177±39	0.06	204±39	181±52	0.12
Triglycerides (mg/dL) (mean±SD)	183±120	142±71	0.05	155±96	181±142	0.40	146±67	161±76	0.50
LDL Cholesterol (mg/dL) (mean±SD)	117±22	100±31	<0.01	117±35	102±40	0.10	119±31	100±40	0.10
HDL Cholesterol (mg/dL) (mean±SD)	44±11	39±9	0.03	47±12	42±9	0.09	56±21	49±16	0.24
HbA1c (%) (mean±SD)	5.4±0.4	5.5±0.5	0.30	5.8±1.1	5.7±0.8	0.92	5.6±0.6	5.6±0.6	0.81
Creatinine (mg/dL) (%) (mean±SD)	1.0±0.1	1.1±0.1	0.11	1.1±0.1	1.1±0.2	0.89	0.8±0.2	1.0±0.5	0.12
Hematocrit (%) (mean±SD)	44±5	43±3	0.15	43±2	43±3	0.89	41±3	40±2	0.03
White blood cell count (10^9^/L) (mean±SD)	5.6±1.3	6.2±2.1	0.06	5.9±1.7	6.4±1.9	0.27	6.7±2.3	6.9±2.1	0.51

Clinical laboratory parameters were available for all subjects (table 1). Hematocrit and white blood cell counts were not significantly different. Total cholesterol and LDL-cholesterol levels were significantly lower in the coronary artery disease group, probably reflecting a higher use of statins.

### Prediction of Coronary Disease Using Risk Factors and Biochemical Markers

Traditional risk factors, including body weight, smoking, and systolic and diastolic blood pressure did not correlate significantly with the extent of coronary disease in a rank correlation analysis. Total cholesterol (but not LDL-cholesterol) level was found to be inversely related with the CAD-index (rho = −0.41, P<0.0001), which may in part reflect the higher use of statins and better blood lipid control in cases. In addition, other parameters were found to positively (potassium, blood urea nitrogen, phosphorus and osmolarity) or negatively (calcium and HDL-cholesterol) correlate with CAD-index (rho>0.2). Of note, important clinical markers such as LDL-C (rho = 0.02), CRP (rho = −0.12) and homocysteine (rho = 0.02) exhibited a poor correlation with CAD-index, which could result from treatment of affected individuals. In a multivariate correlation analysis, the combination of risk factors and biochemical markers only poorly predicted the extent of coronary artery disease (r^2^ = 0.228).

### Gene Expression

Gene expression data from 222 out of 241 subjects were available for this analysis (110/121 cases and 112/120 controls); RNA from the remaining 19 subjects did not pass quality control due to degradation.

In a univariate analysis, 160 genes were found to correlate with CAD-Index with an absolute rank correlation coefficient (rho) >0.2 (*P*<0.003). All probesets correlating with CAD-Index are listed in table 2. Most of these genes are known to be involved in hematopoietic cell differentiation, cell growth or growth arrest, apoptosis, cell adhesion, matrix modulation and inflammatory and immune response, processes known to modulate atherosclerosis.

**Table 2:** List of 160-gene model predictive of the extent of coronary artery disease.

Symbol	U133A ID	95Av2 ID	Name	Pathway	Rho
*AIF1*	207823_s_at	37011_at 33641_g_at	allograft inflammatory factor 1	Angiogenesis	0.21
		3640_at 37764_at		Inflammatory response	
*MMP19°*	204575_s_at		matrix metalloproteinase 19	Angiogenesis	0.22
				Response to metal ion	
				Extracellular matrix modulation	
*EPIM*	207346_at		epimorphin	Angiogenesis	0.22
MMP24°°	78047_s_at		Matrix metalloproteinase 24 (membrane-inserted)	Angiogenesis	0.20
				Response to metal ion	
				Extracellular matrix modulation	
*CRADD°*	209833_at	822_s_at 1211_s_at	CASP2 and RIPK1 domain containing adaptor with death domain	Apoptosis	0.25
*WDR13°*	222138_s_at	727_at	WD repeat domain 13	Apoptosis	0.24
*PDE4D*	210837_s_at	38526_at	phosphodiesterase 4D, cAMP-specific (phosphodiesterase E3 dunce homolog, Drosophila)	Apoptosis	0.22
*AK2°°*	212174_at	40789_at 40788_at	adenylate kinase 2	Apoptosis	0.28
*FOLH1°*	217487_x_at	1740_g_at 1739_at 1655_s_at	folate hydrolase (prostate-specific membrane antigen) 1	Apoptosis	0.22
*TGM5*	207911_s_at	33001_s_at	transglutaminase 5	Apoptosis	0.24
*P53AIP1°*	220403_s_at		p53-regulated apoptosis-inducing protein 1	Apoptosis	0.25
*NALP1*	211822_s_at		NACHT, leucine rich repeat and PYD (pyrin domain) containing 1	Apoptosis	0.26
				Inflammatory response	
*LGALS9°*	203236_s_at	766_at 38091_at	galectin 9	Cell adhesion	0.25
*ICAM1°*	202637_s_at	32640_at	intercellular adhesion molecule 1 (CD54), human rhinovirus receptor	Cell adhesion	0.21
PCDHGC3	205717_x_at	657_at 35609_at 1691_at 1690_at 1169_at	protocadherin gamma subfamily C, 3	Cell adhesion	0.20
GPLD1	206265_s_at	934_at 1293_s_at	glycosylphosphatidylinositol specific phospholipase D1	Cell adhesion	0.21
				T/B cell proliferation	
*CDH11°*	207173_x_at	36976_at 2087_s_at	cadherin 11, type 2, OB-cadherin (osteoblast)	Cell adhesion	0.24
*DSC3*	206032_at	32417_at	desmocollin 3	Cell adhesion	0.20
				Cytoskeleton	
*LAMB3°*	209270_at	36929_at	laminin, beta 3	Cell adhesion	0.25
*PKP4*	214874_at	33475_at	plakophilin 4	Cell adhesion	0.22
				Cytoskeleton	
*FN1*	214702_at		Fibronectin 1	Cell adhesion	0.21
*IIp45°*	48659_at		IGFBP-2-Binding Protein, IIp45 (FLJ12438)	Cell adhesion	0.22
*PINK1°°*	209018_s_at	35361_at	PTEN induced putative kinase 1	Cell growth & growth arrest	0.22
				Apoptosis	
*FKBP8°°*	40850_at	40850_at	FK506 binding protein 8, 38kDa	Cell growth & growth arrest	0.25
*UBXD1°°*	220757_s_at		UBX domain-containing protein 1	Cell growth & growth arrest	0.21
RXRA°	202426_s_at	405_at 32800_at	retinoid X receptor, alpha	Cell growth & growth arrest	0.24
				Apoptosis	
*RIS1°*	213338_at	35692_at	Ras-induced senescence 1	Cell growth & growth arrest	0.28
*NFYC°*	202215_s_at	40466_at	nuclear transcription factor Y, gamma	Cell growth & growth arrest	0.30
*CLN3*	209275_s_at	497_at	ceroid-lipofuscinosis, neuronal 3, juvenile (Batten, Spielmeyer-Vogt disease)	Cell growth & growth arrest	0.27
				Apoptosis	
*RARA*	211605_s_at	1337_s_at	retinoic acid receptor, alpha	Cell growth & growth arrest	0.26
HCFC1	202473_x_at	37910_at	host cell factor C1 (VP16-accessory protein)	Cell growth & growth arrest	0.23
*PSG3*	203399_x_at	40857_f_at	pregnancy specific beta-1-glycoprotein 3	Cell growth & growth arrest	0.22
*STAU2°*	204226_at	38341_at 32386_at	staufen, RNA binding protein, homolog 2 (Drosophila)	Cell growth & growth arrest	0.26
*ELAVL2*	208427_s_at	36411_s_at 36410_f_at	ELAV (embryonic lethal, abnormal vision, Drosophila)-like 2 (Hu antigen B)	Cell growth & growth arrest	0.25
*TP53I11°*	214667_s_at	36136_at	tumor protein p53 inducible protein 11	Cell growth & growth arrest	0.31
NPR3	219789_at	34519_at	natriuretic peptide receptor C/guanylate cyclase C (atrionatriuretic peptide receptor C)	Cell growth & growth arrest	0.21
				Angiogenesis	
*PTP4A1°*	200730_s_at	843_at 33413_at	protein tyrosine phosphatase type IVA, member 1	Cell growth & growth arrest	0.27
*STC2*	203439_s_at	32043_at	stanniocalcin 2	Cell growth & growth arrest	0.24
				Response to metal ion	
*SEMA3C*	203788_s_at	377_g_at 376_at	sema domain, immunoglobulin domain (Ig), short basic domain, secreted, (semaphorin) 3C	Cell growth & growth arrest	0.28
				Immune response	
*CCNA1*	205899_at	1914_at	cyclin A1	Cell growth & growth arrest	0.20
*PTPRR°*	206084_at	1658_g_at 1657_at	protein tyrosine phosphatase, receptor type, R	Cell growth & growth arrest	0.23
*LHX2*	206140_at	40528_at	LIM homeobox 2	Cell growth & growth arrest	0.21
				T/B cell proliferation	
*CPSF4*	206688_s_at	35743_at	cleavage and polyadenylation specific factor 4, 30kDa	Cell growth & growth arrest	0.22
				Inflammatory response	
*I–4°*	207377_at	31735_at	type 1 protein phosphatase inhibitor	Cell growth & growth arrest	0.21
*MEIS2*	207480_s_at	41388_at	Meis1, myeloid ecotropic viral integration site 1 homolog 2 (mouse)	Cell growth & growth arrest	0.20
*NF2°*	211092_s_at	38007_at 1894_f_at	neurofibromin 2 (bilateral acoustic neuroma)	Cell growth & growth arrest	0.23
				Cytoskeleton	
*BRRN1°*	212949_at	41639_at	barren homolog (Drosophila)	Cell growth & growth arrest	0.32
*CDC42*	214230_at	960_g_at 959_at 39736_at	cell division cycle 42 (GTP binding protein, 25kDa)	Cell growth & growth arrest	0.23
*ZMYND10°*	216663_s_at	32993_s_at	zinc finger, MYND domain containing 10	Cell growth & growth arrest	0.25
*CROC4*	222301_at	40483_at 40482_s_at	transcriptional activator of the c-fos promoter	Cell growth & growth arrest	0.22
*PPP2R5B°*	635_s_at	635_s_at	protein phosphatase 2, regulatory subunit B (B56), beta isoform	Cell growth & growth arrest	0.20
*TRIM45°*	219923_at		tripartite motif-containing protein 45	Cell growth & growth arrest	0.23
*TDRKH*	221053_s_at		tudor and KH domain containing	Cell growth & growth arrest	0.26
*PB1°*	221212_x_at		polybromo 1	Cell growth & growth arrest	0.25
*NEIL1*	219396_s_at		nei endonuclease VIII-like 1	Cell growth & growth arrest	0.31
*PMS2L5°*	179_at		Postmeiotic segregation increased 2-like 5	Cell growth & growth arrest	0.24
*BLOC1S1°*	202592_at		Biogenesis of lysosome-related organelles complex-1, subunit 1	Cell growth & growth arrest	0.23
*BRF2°*	218955_at		subunit of RNA polymerase III transcription initiation factor, BRF1-like	Cell growth & growth arrest	0.23
*ASNA1°°*	202024_at		Arsenical pump-driving ATPase	Cell growth & growth arrest	0.21
*SIRT5°°*	221010_s_at		sirtuin (silent mating type information regulation 2 homolog) 5	Cell growth & growth arrest	0.21
*HIST1H4G*	208551_at		histone 1, H4g	Cell growth & growth arrest	0.27
*SLD5*	211767_at		SLD5 homolog	Cell growth & growth arrest	0.27
MAN2A2°	202032_s_at	41766_at 38188_s_at	mannosidase, alpha, class 2A, member 2	Cell-cell interaction	0.28
*GJB3°*	215243_s_at	41076_at	gap junction protein, beta 3, 31kDa (connexin 31)	Cell-cell interaction	0.24
*PLXNA2*	207290_at	40395_at	plexin A2	Cell-cell interaction	0.21
*ADH1B*	209613_s_at	35730_at	alcohol dehydrogenase IB (class I), beta polypeptide	Cellular metabolism	0.22
				Immune response	
*FGA*	205650_s_at	38825_at	fibrinogen, A alpha polypeptide	Coagulation	0.26
				Cell adhesion	
				Inflammatory response	
*SERPINB8°*	206034_at	36312_at	serine (or cysteine) proteinase inhibitor, clade B (ovalbumin), member 8	Coagulation	0.23
				Inflammatory response	
*TUBA3°*	212639_x_at	32272_at	tubulin alpha 3	Cytoskeleton	0.28
				Apoptosis	
*ADD1°*	214726_x_at	32146_s_at 32145_at	adducin 1 (alpha)	Cytoskeleton	0.21
ARHGAP4	204425_at	39649_at	Rho GTPase activating protein 4	Cytoskeleton	0.23
LLGL1°°	206123_at	804_s_at 33200_at	lethal giant larvae homolog 1 (Drosophila)	Cytoskeleton	0.22
				Cell growth & growth arrest	
*KRT6A*	209125_at	39016_r_at 39015_f_at	keratin 6A	Cytoskeleton	0.28
*SMPX°*	219772_s_at		small muscle protein, X-linked	Cytoskeleton	0.20
*STXBP2°°*	209367_at	38259_at	syntaxin binding protein 2	Cytoskeleton - Exocytosis	0.25
PLAUR°°	211924_s_at	189_s_at	plasminogen activator, urokinase receptor	Extracellular matrix modulation	0.20
				Inflammatory response	
*COL13A1°*	211809_x_at		collagen, type XIII, alpha 1	Extracellular matrix modulation	0.25
*ABCC6*	214033_at		Up-regulated gene 7	Extracellular matrix modulation	0.26
*ITPK1°°*	210740_s_at	35755_at	inositol 1,3,4-triphosphate 5/6 kinase	Hematopoietic cell differentation	0.30
*FTL°*	212788_x_at	35083_at	ferritin, light polypeptide	Hematopoietic cell differentation	0.24
				Response to metal ion	
*FANCC°*	205189_s_at	35713_at 160034_s_at	Fanconi anemia, complementation group C	Hematopoietic cell differentation	0.23
				Cell growth & growth arrest	
				Inflammatory response	
				Apoptosis	
SMAD5	205187_at	39926_at 1952_s_at 1013_at	SMAD, mothers against DPP homolog 5 (Drosophila)	Hematopoietic cell differentation	0.22
*NOTCH2°*	202445_s_at	38083_at	Notch homolog 2 (Drosophila)	Hematopoietic cell differentation	0.20
				Angiogenesis	
*GATA1°*	210446_at	36787_at	GATA binding protein 1 (globin transcription factor 1)	Hematopoietic cell differentation	0.25
*IL9R°*	217212_s_at 208164_s_at	938_at	interleukin 9 receptor	Hematopoietic cell differentation	0.20 0.24
*CRSP2°*	202612_s_at		Cofactor required for Sp1 transcriptional activation subunit 2	Hematopoietic cell differentation	0.24
*NOX4*	219773_at		NADPH oxidase 4	Hematopoietic cell differentation	0.23
*L3MBTL°*	206822_s_at		Lethal(3)malignant brain tumor-like protein	Hematopoietic cell differentation	0.32
*RNF24*	210706_s_at		Ring finger 24	Hematopoietic cell differentation	0.28
*KLF3°°*	219657_s_at		Kruppel-like factor 3 (basic)	Hematopoietic cell differentation	0.21
*MARCH2°°*	210075_at	39910_at	membrane-associated ring finger (C3HC4) 2	Immune response	0.24
*GPSM3°*	214847_s_at	39049_at	G-protein signalling modulator 3 (AGS3-like, C. elegans)	Immune response	0.27
*IGSF4B*	213948_x_at	39288_at	immunoglobulin superfamily, member 4B	Immune response	0.25
*PRG3°*	220811_at		proteoglycan 3	Immune response	0.24
*AGPAT1°*	32836_at	32836_at	1-acylglycerol-3-phosphate O-acyltransferase 1 (lysophosphatidic acid acyltransferase, alpha)	Inflammatory response	0.29
				Lipid biosynthesis	
*EPHB2°*	211165_x_at	902_at 41678_at 2088_s_at	EPH receptor B2	Inflammatory response	0.22
				Cell-cell interaction	
PRKAR1B°	212559_at	1091_at	protein kinase, cAMP-dependent, regulatory, type I, beta	Inflammatory response	0.25
				T/B cell proliferation	
*PAFAH1B1°*	200815_s_at	32569_at	platelet-activating factor acetylhydrolase, isoform Ib, alpha subunit 45kDa	Inflammatory response	0.27
*NFX1*	202585_s_at	34667_at	nuclear transcription factor, X-box binding 1	Inflammatory response	0.26
KCNMB1°°	209948_at	38298_at	potassium large conductance calcium-activated channel, subfamily M, beta member 1	Ion channel	0.22
*CHRNA5*	206533_at	36397_at	cholinergic receptor, nicotinic, alpha polypeptide 5	Ion channel	0.21
				Neurotransmission	
*SAH°*	210377_at	33279_s_at 33278_at	SA hypertension-associated homolog (rat)	Lipid metabolism	0.30
*HLCS*	207833_s_at	37764_at	holocarboxylase synthetase (biotin-[proprionyl-Coenzyme A-carboxylase (ATP-hydrolysing)] ligase)	Metabolic homeostasis	0.21
*MAOA*	204388_s_at	41772_at 41771_g_at 41770_at	monoamine oxidase A	Neurotransmission	0.28
*GABRA6°*	207182_at	34025_at	gamma-aminobutyric acid (GABA) A receptor, alpha 6	Neurotransmission	0.23
*CEPBA°°*	204039_at	32550_r_at	CCAAT/enhancer binding protein (C/EBP), alpha	Progenitor cell differentiation	0.35
				Cell growth & growth arrest	
*SOX4°*	201416_at	33131_at	SRY (sex determining region Y)-box 4	Progenitor cell differentiation	0.20
*ZNF305*	206507_at	37083_s_at 37082_at	zinc finger protein 305	Progenitor cell differentiation	0.21
*ZNFN1A2°*	220567_at		zinc finger protein, subfamily 1A, 2	Progenitor cell differentiation	0.23
*ZNF3°*	219605_at		zinc finger protein 3 (A8–51)	Progenitor cell differentiation	0.22
				Response to metal ion	
				Immune response	
*CAPN5°*	205166_at	38504_at	calpain 5	Response to injury	0.25
				Cell growth & growth arrest	
*MTF1*	205323_s_at	38945_at	metal-regulatory transcription factor 1	Response to metal ion	0.25
CA12°	203963_at	36454_at	carbonic anhydrase XII	Response to metal ion	0.26
*NEDD4L°*	212445_s_at	39356_at	neural precursor cell expressed, developmentally down-regulated 4-like	Response to metal ion	0.22
CABIN1°	202624_s_at	37652_at	calcineurin binding protein 1	T/B cell proliferation	0.21
*SH3BP2°*	209370_s_at	1303_at	SH3-domain binding protein 2	T/B cell proliferation	0.23
				Immune response	
TNFRSF5	35150_at	35150_at 35149_at	tumor necrosis factor receptor superfamily, member 5	T/B cell proliferation	0.22
				Inflammatory response	
				Immune response	
*PBX2°°*	211097_s_at	38295_at	pre-B-cell leukemia transcription factor 2	T/B cell proliferation	0.25
*IL2*	207849_at		interleukin 2	T/B cell proliferation	0.22
				Cell growth & growth arrest	
				Immune response	
*HDAC5°°*	202455_at		histone deacetylase 5	T/B cell proliferation	0.28
				Inflammatory response	
PIP°	206509_at	41094_at 325_s_at	prolactin-induced protein	T/B cell regulation	0.23
*GAD2°*	216651_s_at	32280_at 32279_at	glutamate decarboxylase 2 (pancreatic islets and brain, 65kDa)	T/B cell regulation	0.21
*PNPLA2°°*	39854_r_at	39854_r_at	patatin-like phospholipase domain containing 2	Triglyceride homeostasis	0.24
MGLL°	211026_s_at	35792_at	monoglyceride lipase	Triglyceride homeostasis	0.27
*MJD*	216657_at	36819_at	Machado-Joseph disease (spinocerebellar ataxia 3, olivopontocerebellar ataxia 3, autosomal dominant, ataxin 3)	Ubiquitination	0.2
*FBXO31*	219784_at		F-box only protein 31	Ubiquitylation	0.26
GGA3°	211815_s_at	37959_at	golgi associated, gamma adaptin ear containing, ARF binding protein 3	Ubiquitylation	0.26
*N4BP1°*	48612_at		Nedd4 binding protein 1	Ubiquitylation	0.21
*BC002942*	31837_at	31837_at	hypothetical protein BC002942		0.28
*MGC21416°*	212340_at	37891_a	hypothetical protein MGC21416		0.27
*DKFZp586F1822*	37891_at		DKFZp586F1822		
*CDRT1*	215999_at	31781_at	CMT1A duplicated region transcript 1		0.23
*KIAA0241°*	212475_at	39761_at	KIAA0241 protein		0.28
*13CDNA73°*	214319_at	33276_at	hypothetical protein CG003		0.24
*SH3TC1°*	219256_s_at		SH3 domain and tetratricopeptide repeats 1		0.28
*RER1°*	213114_at		RER1 homolog (S. cerevisiae)		0.26
*DKFZp564M0616*	215763_at		cDNA DKFZp564M0616		0.26
*KIAA0882°*	212960_at		KIAA0882		0.22
*FLJ11155*	219750_at		hypothetical protein FLJ11155		0.23
*LOC51145*	220752_at		erythrocyte transmembrane protein		0.23
*SEC61A2*	219499_at		Protein transport protein Sec61 alpha subunit 2		0.20
*C12orf4°*	218374_s_at		chromosome 12 open reading frame 4		0.24
*STYK1*	221696_s_at		serine/threonine/tyrosine kinase 1		0.22
*FLJ20674*	220137_at		hypothetical protein FLJ20674		0.24
*PRO1693*	221137_at		PRO1693		0.22
*FLJ12058°°*	215971_at		FLJ12058 fis, clone HEMBB1002092		0.21
*FLJ10305°*	216501_at		hypothetical protein FLJ10305		0.25
*DKFZP434O047*	208008_at		DKFZP434O047 protein		0.25
*FLJ10970*	219230_at		hypothetical protein FLJ10970		0.21
*FLJ22209*	216450_x_at		cDNA: FLJ22209 fis, clone HRC01496		0.20
*FLJ11996*	207487_at		hypothetical protein FLJ11996		0.24
*FLJ20241°*	207083_s_at		putative NFkB activating protein		0.24
*FLJ14220°*	219310_at		FLJ14220, chromosome 20 open reading frame 39		0.21
*SYNGR1°*	213854_at		synaptogyrin 1		0.22
*FLJ11871°*	220915_s_at		FLJ11871, DKFZp686I0814		0.24
*LRCH4*	204692_at		Leucine rich repeat neuronal 4		0.26
*PRO2533*	220787_at				0.23
*EST*	222308_x_at				0.24
*EST*	222302_at				0.22
*EST*	215906_at				0.28

The 8 most predictive genes according to the VIP analysis are listed in bold; °° indicated the 19 genes and °or°° the 90 genes that were found to be differentially expressed in a multiway ANOVA.

Using log-transformed data with signal intensities >80, only 19 probesets were found to be significantly differentially expressed in a multiway ANOVA (smoking, age, gender, cohort, race, CAD (i.e. case vs. control) and CAD-index as fixed factors or random effects, respectively) (table 2). However, when only taking the 20 controls with the least predicted CAD versus the 20 cases with the most predicted disease into account, a formal comparison yielded 90 out of the 160 probesets with statistically significant differential expression (p<0.05, no adjustment for multiple comparisons) (table 2). rt-PCR confirmed the Affymetrix results for 7 of the 8 genes tested in 20 cases and 20 controls (FKBP8, ITPK1, MARCH2, PNPLA2, TUBA3, UBXD1, FTL); the remaining gene (PINK1) did not show a significantly different expression on rt-PCR.

### Correlation of Gene Expression Profile with Coronary Disease

**Figure 1:**
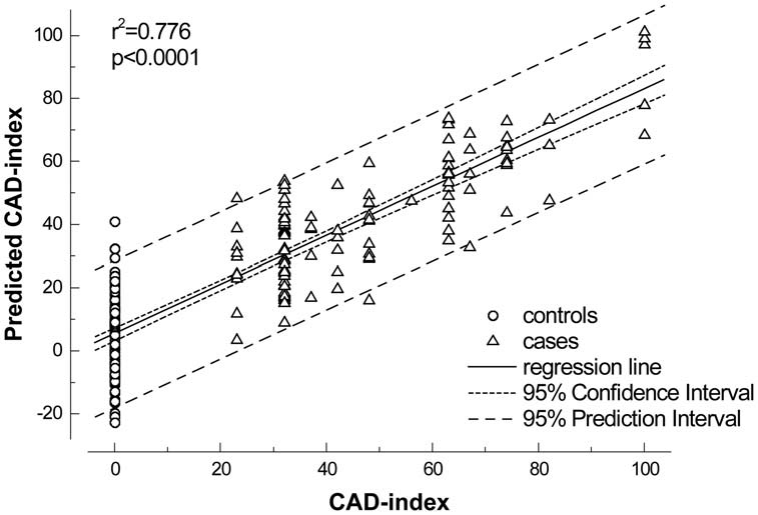
Partial least squares plot of nominal CAD index versus predicted CAD index. Result of the partial least squares analysis including all controls and all cases; n = 222 and 160 genes. Cases are represented as triangles and controls as circles. The CAD-index as predicted by the gene expression is plotted versus the nominal CAD-index as obtained from coronary angiography. Regression line of the predicted CAD index versus nominal CAD-Index is displayed by the full line including 95% confidence interval of the regression (dotted lines) and the 95% prediction interval (striped lines). Goodness of fit is indicated by r^2^ = 0.776 (p<0.001).

All 160 genes with rho>0.2 were included in the PLS analysis, with CAD-Index as the only response variable. Polynomial regression analysis of the resulting t1-scores versus CAD-Index resulted in the prediction model including 95% confidence range of the regression and the 95% prediction interval with r^2^ = 0.764 (p<0.001) (figure 1). Predictive accuracy was found to be excellent in the overall population (RMSEE (root mean square error of estimation)  = 0.323), but improved with increasing threshold of CAD (RMSEE = 0.249 for controls vs cases with CAD>40; RMSEE = 0.204 for controls vs cases with CAD>60 and RMSEE = 0.172 for controls vs cases with CAD>70).

**Figure 2:**
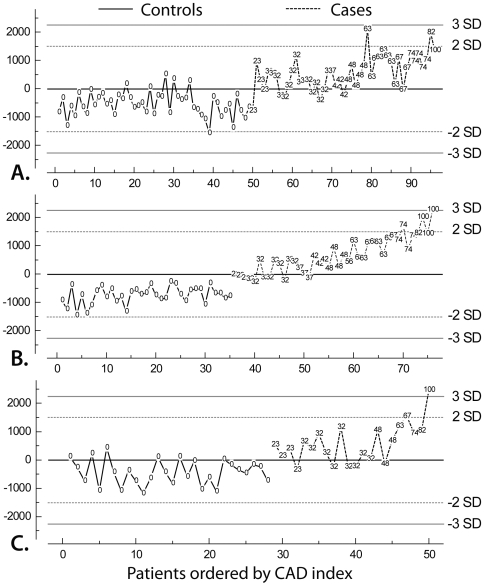
Partial least squares plot per cohort. Results of the partial least squares regression analysis with 160 genes applied separately to each of the three cohorts (A) “Matched Men” (B) “Unmatched Men” and (C) ‘“Unmatched Women”. Models were each time constructed in two cohorts and then tested in the third cohort. Individual patients are ordered by their CAD-Index. Labels represent the individual CAD-Index. Controls (full line) have all CAD-Index 0, and the CAD-Index of cases (dotted line) increases from 23 up to 100. While the controls remain quite stable in the range of -2 standard deviations, the t1-scores increase with increasing CAD-Index (t1 indicates the t1 score vector result from the PLS analysis).

In order to test for robustness of the model, the PLS analysis was performed separately for each of the three cohorts, with the model repeatedly constructed using two cohorts (training sample) and tested in the third cohort (test sample). While the controls remain quite stable in the range of -2 standard deviations, the t1-scores of the cases were located mainly in the +2 standard deviation range and increase with increasing CAD-Index (figure 2). This relationship is clearly present in each cohort. Cross-validation of the model was also performed by dividing the data into 7 groups of on average 32 subjects and then developing a number of parallel models from reduced data with one of the groups deleted. The omitted group was then used as a test data set, and the differences between actual and predicted CAD-Indices were subsequently calculated for these data points. The reduced models validation demonstrated a Q^2^cum of 0.776, indicating an excellent predictive ability.

**Figure 3:**
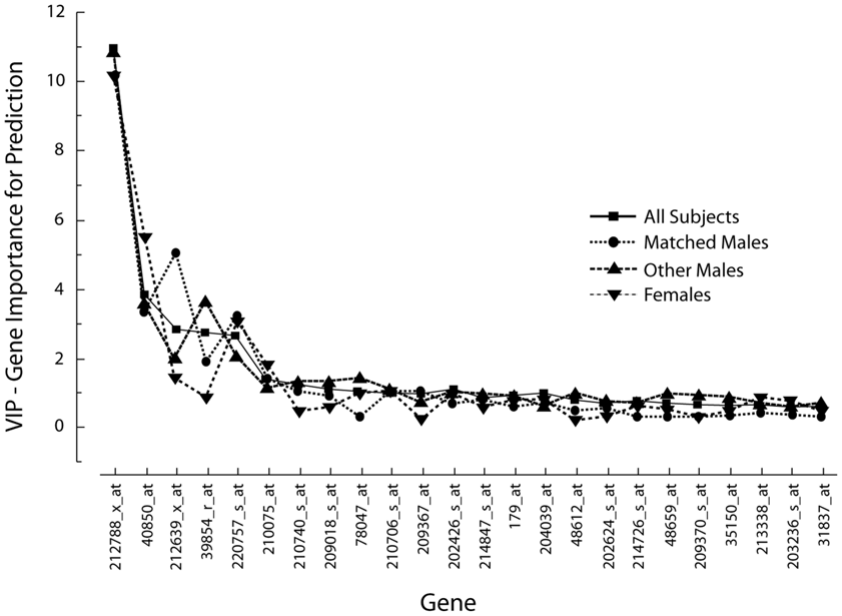
VIP. Variable Importance in the Projection (VIP) for the separate PLS analyses of the three different cohorts compared to the PLS analysis including all subjects. Displayed are the 24 probesets with the highest VIP. The curve shows a steep decrease for the first 8 genes (listed in table 2); the contribution of further genes is comparable as suggested by almost linear curves.

A Variable Importance in the Projection (VIP) of each gene for the separate PLS analyses of the three cohorts compared to the PLS analysis including all subjects was calculated. The VIP of the first 24 genes shows only little variation between the three cohorts suggesting a rather high stability of the prediction model (figure 3). A set of eight genes appears to have the highest impact on the model (*FTL*, *FKBP8*, *TUBA3*, *PNPLA2*, *UBXD1*, *MARCH2*, *ITPK1*, *PINK1*, in order of contribution; listed in bold in table 2). A PLS analysis only involving these eight highest ranking genes in the VIP analysis showed that the expressions profiles of these eight genes are also able to predict the CAD-Index (r^2^ = 0.752). Adding traditional risk factors and biochemical markers do not significantly improve this model (r^2^ = 0.782).

### Test of Predictability in Human Aorta Tissue Samples

**Figure 4:**
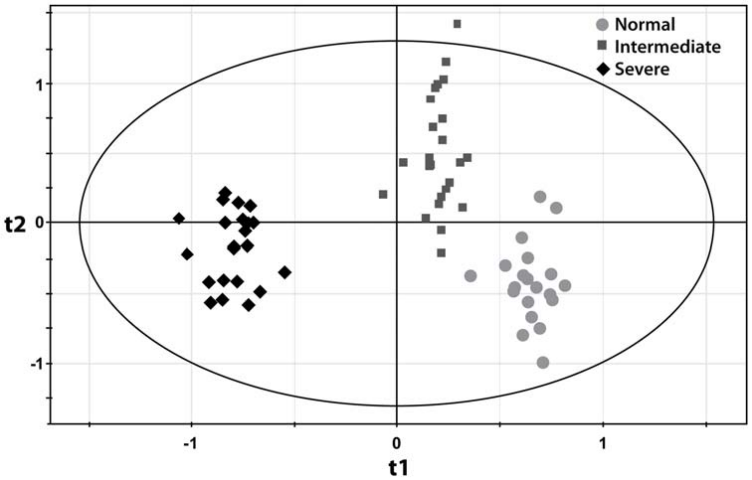
Partial least squares discriminant analysis in atherosclerotic aortas. Result of the partial least squares discriminant analysis (t1/t2 score plot) including all aorta samples; n = 67. Dots represent normal aortas, squares represent intermediate atherosclerosis and diamonds indicate severe aorta atherosclerosis. Using expression data in aorta samples, the PLS analysis using the 160 peripheral blood genes adequately separates normal aortas from intermediate and severe atherosclerotic aortas (the ellipse indicates Hotelling's T2 95% confidence region; t1 and t2 indicate the t1 and t2 score vector results from the PLS-DA analysis).

Since the genes whose expression contributes to prediction of CAD were studied within circulating leukocytes, we sought to define whether they actually reflect a molecular process that is ongoing within atherosclerotic arteries or not. Furthermore, as a test of reproducibility of the contribution of these 160 genes to predicting atherosclerotic disorders, we have investigated whether the in situ expression pattern of our 160 genes derived from peripheral blood could also adequately predict the severity of aorta atherosclerotic lesions. To achieve this goal, we have used gene expression data extracted from a large set of human aortas obtained from heart donors (n = 67), an independent human model of atherosclerosis. Excluding genes that are not present on the microarray used in the aorta expression study, the expression pattern of the remaining genes accurately separated the aorta samples according to the severity of atherosclerosis (figure 4). These results indicate that gene expression changes in peripheral blood are correlated with the extent of coronary atherosclerosis reflect similar pathophysiological changes in atherosclerotic arteries.

## Discussion

In this large-scale expression analysis of peripheral whole blood cells, we have found 160 genes whose expression correlates with the severity of angiographically documented coronary artery atherosclerosis. Taking into account that the CAD-Index is a semi-quantitative estimate of the extent of coronary atherosclerotic disease, which implies variation across subjects even with the same degree of disease, the prediction based on expression pattern of these genes is robust. Our findings are also robust as assessed by internal validation and consistency across three distinct subgroups. Importantly, the in situ expression pattern of the 160 genes derived from the peripheral blood analysis was also predictive of the severity of atherosclerosis in human aorta tissue. This provides validation of the association of this set of genes with atherosclerosis and support for the concept that peripheral blood gene expression reflects pathophysiology in the vascular wall. Taken together, the molecular signature in peripheral blood for varying degrees of coronary artery disease is remarkably consistent with that seen in the atherosclerotic arterial wall, providing valuable new information of the pathways and their genes that are involved in the atherosclerotic process.

Peripheral blood is easily accessible and routinely used for diagnostic laboratory analysis and thus is a good resource for additional tests that might define extent of coronary artery disease. Several inflammatory markers, including high sensitivity C-reactive protein (CRP) are associated with cardiovascular risk, independently from traditional risk factors [21]. Nevertheless, there is debate as to the additional prognostic value of these tests beyond traditional risk factors [22]. Other non-invasive analyses, such as coronary multislice CT can identify the extent of coronary artery disease, but such tests require specialized equipment and involve use of intravenous contrast and radiation. A simple blood test that predicts the extent of coronary artery disease could provide an additional useful tool for screening for coronary artery disease in at-risk populations. A similar approach has been successfully used for detection of cardiac allograft rejection and the response to immunosuppressive therapy [23].

Most of the differentially expressed genes in the present study are involved in bone marrow cell differentiation, cell growth or growth arrest, apoptosis, cell adhesion and matrix modulation, and inflammatory and immune response, processes known to modulate atherosclerosis. Since blood samples were taken in stable patients, our finding that circulating blood cells differentially express many pro-inflammatory genes supports the paradigm that inflammation is an important process in patients with coronary artery disease. Expression patterns of the same genes were found to correlate with the extent of atherosclerosis in human aortas as well, indicating that gene expression patterns in peripheral blood cells associated with coronary artery disease to some extent mirror gene expression changes in the atherosclerotic vessel wall. Indeed, many of the genes shared by our predictive models modulate monocyte or macrophage function, including *MAN2A* [24], *RXRA* [25], *LGALS9* [26], *PSG3* [27], *CEPBA* [28], *ARGHAP4* [29], *MADH5* [30], *AIF1* [31], *ELAVL2* [32], *STXBP2* [33], *KCNMB1* [34], *PDE4D* [35], *EPHB2* [36], *GGA3* [37], *PLAUR* [38], *NPR3* [39] and *TNFRSF5* (CD40) [40]. Interestingly, four of these genes (*KCNMB1, NEDD4L, ADD1* and *NPR3*) have been implied in genetic susceptibility for hypertension [41]–[44], while two genes have been associated with genetic susceptibility for stroke (*PDE4D*) [45] or myocardial infarction (*ADD1*) [46]. The present results also appear to support a role for ferritin light chain (*FTL*) in atherosclerosis [47]. Ferritin is the major intracellular iron storage protein that plays a major role in the reaction to oxidative stress. Using a proteomic approach, You et al. found that the levels of ferritin light chain protein were significantly increased in atherosclerotic coronary arteries [48]. Ferritin light chain is also upregulated in circulating leukocytes of patients with juvenile rheumatoid arthritis, sickle cell disease, autoimmune renal disease or multiple sclerosis, indicating that altered *FTL* gene expression in peripheral cells of CAD patients might in at least in part reflect a general pro-inflammatory state that leads to degenerative changes [49]–[52].

We intentionally did not separate peripheral blood cells or leukocyte subtypes. There is currently little pathophysiological evidence that the study of leukocyte subgroups would add to our predictive model and the isolation process could, in itself, affect the gene expression pattern. Using whole blood cells not only allows aggregate RNA expression analysis per patient without the need to pool rare subtypes, but is also more practical from a clinical perspective. Leukocyte levels in all groups were very similar, although it cannot be excluded that the percentage of specific subtypes differ between groups, and hence that different numbers of subtypes are responsible for the observed effect. Peripheral whole blood might also include differential expression signatures from reticulocytes, platelets or rare hematopoietic progenitors.

In a recent paper, Wingrove et al reported 526 differentially expressed genes (>1.3-fold expression) from a genome-wide microarray analysis of peripheral blood mononuclear cells of 27 cases with angiographically documented CAD and 14 controls [15]. The authors found that 14 genes, out of a a set of 106 genes including the 50 most significant genes from the microarray analysis and 56 genes selected from the literature, were associated with the presence of CAD and the severity of CAD in two independent cohorts. The overlap between our study and the Wingrove study at the individual gene level appears to be very limited. This might be in part due to the considerably different design of our study. Not only did we prefer a correlation-based approach, the Wingrove study also used a much smaller subset of patients for unbiased microarray-based gene discovery, and added 56 literature-based genes for the subsequent analysis in their two cohorts. As a result of our correlation analysis, we also did not exclude genes with differential expression below 1.3-fold; since atherosclerosis is a chronic disease, small changes in gene expression might accrue over time and result in a clinically relevant phenotype. Moreover, in contrast with our study, a substantial proportion of microarray samples in the Wingrove analysis were taken from patients presenting with an acute coronary syndrome, which might have significantly influenced expression levels. Another reason for the discrepancies between the two studies might be the different types of microarray used and different types of cells studied. In our study, we analyzed RNA from whole blood in all patients, in contrast with isolated mononuclear cells used in the discovery phase of the Wingrove study. An Ingenuity Pathway Analysis (IPA, Ingenuity Systems, Redwood City, Ca; USA) comparing the 366 genes with p<0.05 (from the 526 probesets) and our 160 genes with rho>0.2 shows that similar biological functions were hit, despite the different microarrays and different matrices used (data not shown). In any case, the discrepancies between both studies suggest that these results need to be validated in larger and more diverse populations.

Of the 160 genes we found to be correlated with the extent of CAD, only 19 were significantly differentially expressed between all cases and controls, while gene expression was significantly different for 90 genes when comparing 20 patients with the least predicted CAD-index to 20 patients with the highest predicted CAD-index. Most of our cases only have mild to moderate disease, with only a minority having extensive disease. Thus, in part as a result of our proteomics-driven patient selection, there is likely to be a very gradual transition from controls to cases, with the distrubution of cases being skewed towards the lower end of CAD-index. We therefore assumed that the difference between controls and cases was not likely to be very large, hence our preference for a correlation-based analysis. Furthermore, since the average age of the controls was 52 years, it is highly likely that some degree of coronary atherosclerosis is present in these subjects. Interestingly, patients with normal angiograms but with microvascular dysfunction may also demonstrate peripheral monocyte activation, although not to the extent seen in patients with angiographically documented coronary artery disease [53]. Our findings that the present model also accurately predicts the severity of coronary artery disease in female patients, in whom advanced coronary artery disease is less likely at the age of 50, is reassuring. It is notable that CRP and LDL did not predict disease in our population. However, while these are excellent markers for future cardiovascular events [54], their ability to predict the severity of angiographically documented CAD is known to be low [55]–[58]. We even observed an inverse correlation between LDL-cholesterol levels and CAD-index. This might be at least in part due to differences in treatments, especially in statin use. Statins might indeed blunt gene expression differences in vascular cells and circulating monocytes to certain extent, which might have influenced our findings [59], [60].

In conclusion, the combined predictive value of differentially expressed genes in peripheral blood correlates with the extent of coronary atherosclerosis. Importantly, the expression pattern of the same genes is also correlated with the extent of disease in atherosclerotic aortas. While these findings need prospective validation in further populations, our findings also suggest that gene expression profiles might represent a novel and promising non-invasive test to assess the presence and extent of coronary artery disease. Although the extent of angiographic disease is a strong predictor of clinical outcome, further studies in larger and unselected populations will also be needed to examine the potential role of gene expression patterns in predicting outcome and to address potential confounding factors.
